# Clinical Audit of Driving Information Collection and Advice in NY&Y CAMHS Eating Disorder Service

**DOI:** 10.1192/bjo.2026.11710

**Published:** 2026-06-30

**Authors:** Nihit Gupta, Koralagamage Kavindu Appuhamy, Gareth Howel, Rebecca Feris, Henry Hodgson

**Affiliations:** Tees, Esk and Wear Valleys NHS Foundation Trust, York, United Kingdom

## Abstract

**Aims:**

The aims of this audit are to assess compliance with local audit standards for: (1) documentation of driving status at initial assessment; (2) documentation of DVLA notification advice; and (3) documentation and follow-up of driving safety advice aligned with DVLA/regional guidance because young people with eating disorders (EDs) may experience physical and cognitive impairment (e.g., blackouts, hypotension, hypoglycaemia) that can compromise driving safety. UK Driver and Vehicle Licensing Agency (DVLA) guidance requires individuals to notify DVLA if an ED affects fitness to drive. Yet, no CAMHS-specific local process existed to guide systematic assessment and documentation of the risk that young people pose from driving, to ultimately maintain patient and public safety.

**Methods:**

A retrospective electronic case-note review was undertaken within the North Yorkshire & York CAMHS ED Service. All patients aged ≥15 years 9 months up to October 2024 were screened (n=65); 48 met the inclusion criteria. An agreed proforma captured driving status documentation; presence and content of driving safety advice; how this aligned with DVLA/ NHS guidance; follow-up advice at specified intervals; and explicit documentation of DVLA notification advice.

**Results:**

No cases documented driving status at initial assessment (0/48). Any documented driving safety advice was present in 33.3% (16/48) of cases. Among those with documented advice (n=16), follow-up driving advice was recorded in 93.8% (15/16), and 100% had documented advice aligned with DVLA/NHS guidance (16/16). Explicit documentation that the patient should notify DVLA was present in 43.8% (7/16). The key areas for improvement were the lack of risk assessment regarding driving; documentation at initial assessment; and inconsistent DVLA notification advice.

**Conclusion:**

This audit identified that the ED service was not adequately assessing the driving status of young people with ED and/or providing the necessary advice, which could have implications for patient safety. Where advice was documented, quality and follow-up were high, suggesting that introducing structured prompts would be beneficial.

A revised initial assessment pro forma incorporating a structured “Driving Status & Advice” section and visual reminders (a clinic poster) has been implemented; a co-produced CAMHS-specific ED and Driving patient leaflet and an updated follow-up review template are in progress. The second cycle will evaluate the impact of the changes and the standardisation of DVLA-related documentation across the pathway.

